# Association of Other Autoimmune Diseases With Thyroid Eye Disease

**DOI:** 10.3389/fendo.2021.644200

**Published:** 2021-03-05

**Authors:** Mary Kelada, Parizad Avari, Soma Farag, Rashmi Akishar, Rajni Jain, Ahmad Aziz, Claire Feeney, Vassiliki Bravis, Karim Meeran, Vickie Lee

**Affiliations:** ^1^ Imperial College School of Medicine, Imperial College London, London, United Kingdom; ^2^ Department of Metabolism, Digestion and Reproduction, Imperial College London, London, United Kingdom; ^3^ The Western Eye Hospital, Imperial College Healthcare NHS Trust, London, United Kingdom; ^4^ Department of Ophthalmology, Central Middlesex Hospital, London North West Healthcare NHS Trust, London, United Kingdom

**Keywords:** thyroid eye disease, polyautoimmunity, disease severity, ethnically diverse, clinical activity score, immunosuppression, rheumatoid arthritis, psoriasis

## Abstract

**Background:**

Thyroid eye disease (TED) is a potentially disfiguring and sight-threatening autoimmune (AI) orbitopathy, affecting up to 400,000 people in the UK. There are no accurate early predictors of TED severity. Although polyautoimmunity has been shown to affect AI disease severity, its influence on TED severity has never been investigated. The prevalence of polyautoimmunity among TED patients is also unclear, with discordant results reported in the literature. This study evaluates the prevalence of non-thyroid/“other” AI (OAI) conditions in an ethnically diverse TED cohort and assesses how polyautoimmunity affects TED severity and activity.

**Methods:**

A retrospective study of patients presenting to multidisciplinary TED clinics across three North-West London hospitals between 2011 and 2019. Data collected included: 1) demographics; 2) OAI conditions and management; 3) endocrine management of thyroid dysfunction; 4) details of TED and clinical activity score at presentation.

**Results:**

Two hundred and sixty-seven patients with a median age of 46 (35–54) years were included, 79.4% were female and 55% were Black, Asian and minority ethnic (BAME). Thirty-seven patients (13.9%) had OAI conditions, with rheumatoid arthritis (3.7%), vitiligo (3.0%) and psoriasis (3.0%) among the most prevalent. Of patients with OAI conditions, 43.2% (16/37) required immunosuppression prior to TED onset. Non-immunosuppressed patients with OAI conditions had a significantly higher clinical activity score at presentation than TED-only and previously immunosuppressed patients (p=0.02). No significant differences were observed in thyroid receptor antibody titers between these groups.

**Conclusions:**

This study finds a 13.9% prevalence of OAI conditions among TED patients. Patients with OAI conditions overall have a tendency for more severe and significantly more clinically active TED than those without OAI conditions. Larger, prospective studies are warranted to further evaluate polyautoimmunity as an early predictor of TED severity.

## Introduction

Thyroid eye disease (TED), also known as Graves’ ophthalmopathy/orbitopathy, is a distressing, disfiguring, and potentially sight threatening, autoimmune disease (1). TED affects an estimated 400,000 people in the UK (2) and is the most common extrathyroidal manifestation of Graves’ Disease (GD) (3–5). TED can cause a significant disease burden, causing pain, discomfort, double vision and considerable psychosocial distress to its sufferers (1, 6, 7). Around 5% of TED cases are estimated to progress to sight-threatening disease (8); however, there are currently no accurate early predictors of disease severity.

There is a well-established tendency for distinct autoimmune (AI) conditions to co-exist in patients who have previously been diagnosed with an AI condition, such as autoimmune thyroiditis (i.e. polyautoimmunity) (9–11). Although polyautoimmunity has been shown to affect AI disease severity (12–15), its association with TED severity has never been investigated.

There is also a paucity of medical literature investigating the prevalence of polyautoimmunity among TED patients. Only two studies have investigated the prevalence of non-thyroid AI, or “other AI” (OAI), conditions among TED patients. They estimate the prevalence of OAI conditions to be 8%–19% among TED patients (16, 17). However, both studies were carried out in monoethnic cohorts of Graves’ hyperthyroidism patients only. Given that around 3% of TED patients have autoimmune hypothyroidism (Hashimoto’s thyroiditis) and a further 2% never experience thyroid dysfunction (18), these results may not be applicable to all TED patients.

This study aims to evaluate the presence of non-thyroid AI conditions in an ethnically diverse, metropolitan TED cohort and to assess whether there is an association between the presence of polyautoimmunity and TED severity or clinical activity.

## Methodology

This was a retrospective multi-center patient-cohort study based on patients attending three multidisciplinary thyroid eye disease (MDTED) clinics based in North West London, UK, at Imperial College Healthcare NHS Trust (ICHNT; Charing Cross Hospital CXH, Western Eye Hospital WEH) and Central Middlesex Hospital CMH, London North West Healthcare NHS Trust (LNWH). The audit was registered and approved by the relevant Trust departments.

Clinical records of all patients added to the joint MDT-TED clinic databases at CHX, WEH and CMH hospital sites from January 2011 to November 2019 were evaluated for eligibility. Patients were excluded if they had less than 3 months of follow-up, no TED diagnosis according to clinical/radiological criteria or incomplete clinic notes (i.e. no past medical history stated/no letters available for subsequent visits). Patients who had received ophthalmic or endocrine care outside Imperial or LNWH Trusts were also excluded as their hospital records were not accessible.

Data were collected from the electronic hospital records of eligible patients. Demographic data included: age at first presentation at the MDT-TED clinic, gender, ethnicity, smoking status, and family history of thyroid disease (i.e. primary or secondary relative affected). Details of their endocrine care were collected to include: thyroid diagnosis (GD, Hashimoto’s thyroiditis, Hashitoxicosis, hypothyroidism, euthyroid or other) at presentation, initial thyroid receptor antibody (TRAb) titer and thyroid dysfunction management [medication, thyroidectomy, and/or radioiodine (RAI) therapy].

Data collected on TED included: clinical activity score (CAS) at presentation; the patient’s initial and most severe disease severity classification according to European Group on Graves’ Orbitopathy (EUGOGO) criteria; management {selenium supplements, lubricating eyedrops, immunosuppression [first line treatment with intravenous methylprednisolone (IVMP) and/or second line immunosuppression, urgent or elective orbital decompression surgery, orbital radiotherapy (OR)]}.

Other autoimmune (OAI) conditions were only recorded for patients if the diagnosis had been confirmed by a specialist (for example, dermatologist, rheumatologist or gastroenterologist) following relevant clinical examinations and investigations. Steroid cover and immunomodulatory therapy for OAI were also recorded.

### Statistical Analyses

All data were anonymized for statistical analyses using GraphPad Prism, version 8.0. The Shapiro-Wilks test was used to assess the normality of continuous data. Where data are not normally distributed, continuous variables have been expressed as medians (interquartile range). A Spearman rank correlation was used to evaluate the degree of association between two non-parametric variables (i.e. CAS score and TRAb titers). For groupwise analysis of non-parametric continuous data, a Kruskall-Wallis test was carried out, followed by Mann-Whitney *U* tests. Where more comparisons have been made than there are number of groups, alpha values have been adjusted using Bonferroni’s correction to account for multiple comparisons. Chi squared tests were used to compare two categoric variables between groups.

## Results

### Demographics

Two hundred and sixty-seven patients were included in the study. On average, patients were seen within 1.8 (0.8–2.9) months of referral from endocrine clinic to MDT-TED. Patients had a median age of 46 (35–54) years. Of these, 92.5% (247/267) had Graves hyperthyroidism, 3.7% (10/267) had Hashitoxicosis, 3.0% (8/267) had no thyroid hormonal dysfunction and 0.7% (2/267) had other thyroid pathologies, namely thyroid hormone resistance and follicular carcinoma of the thyroid. 79.4% were female and over half the participants (55%) were Black, Asian or minority ethnic (BAME). 13.9% (37/267) patients were noted to have OAI conditions, of whom 21.7% (8/37) had more than OAI condition. Nearly half [43% (16/37)] of patients with OAI conditions required immunosuppressive therapy prior to TED symptom onset. 42.6% (98/267) of patients were euthyroid at TED onset.

### OAI Conditions

The most commonly observed AI condition was rheumatoid arthritis (RA) with the highest prevalence among Caucasian TED patients, (six compared to two BAME patients; **Table 1**). 3.0% (eight patients) had vitiligo and the same for psoriasis. Seven of the eight vitiligo patients were BAME, so this was the most prevalent OAI condition among BAME patients. 1.5% (4/267) had relapsing-remitting multiple sclerosis (RRMS). Overall, we did not detect statistically significant differences in the distribution of OAI conditions among Caucasian and BAME patients (*p*>0.05, post-hoc analysis not displayed).

**Table 1:** Prevalence of non-thyroid autoimmune conditions in patients grouped by ethnicity.

	Ethnicity			Total (n= 267)
	Caucasian (n = 74)	BAME (n = 147)	Not Stated (n = 46)	
**Non-thyroid Autoimmune Conditions**				
* Rheumatoid Arthritis*	6 (8.1%)	2 (1.4%)	2 (4.3%)	10 (3.7%)
* Vitiligo*	1 (1.3%)	7 (4.8%)	0 (0%)	8 (3.0%)
* Psoriasis*	2 (2.8%)	3 (2.0%)	3 (6.5%)	8 (3.0%)
* Relapsing-Remitting Multiple Sclerosis*	1 (1.3%)	2 (1.4%)	1 (2.2%)	4 (1.5%)
* Systemic Lupus Erythematosus*	0 (0%)	2 (1.4%)	0 (0%)	2 (0.7%)
* Crohn's Disease*	1 (1.3%)	1 (0.7%)	0 (0%)	2 (0.7%)
* Type 1 Diabetes Mellitus*	1 (1.3%)	1 (0.7%)	0 (0%)	2 (0.7%)
* Juvenile Idiopathic Arthritis*	0 (0%)	1 (0.7%)	0 (0%)	1 (0.4%)
* Alopecia Areata*	1 (1.3%)	0 (0%)	0 (0%)	1 (0.4%)
* Giant Cell Arteritis*	1 (1.3%)	0 (0%)	0 (0%)	1 (0.4%)
* Polymyalgia Rheumatica*	1 (1.3%)	0 (0%)	0 (0%)	1 (0.4%)
* Sjogren's Disease*	0 (0%)	2 (1.4%)	0 (0%)	2 (0.7%)
* Sarcoidosis*	1 (1.3%)	0 (0%)	0 (0%)	1 (0.4%)
* AI Pancreatitis*	0 (0%)	1 (0.7%)	0 (0%)	1 (0.4%)
* AI Neutropenia*	1 (1.3%)	0 (0%)	0 (0%)	1 (0.4%)
* AI Pernicious Anemia*	0 (0%)	1 (0.7%)	0 (0%)	1 (0.4%)
* pANCA Vasculitis*	0 (0%)	0 (0%)	1 (2.2%)	1 (0.4%)
* Coeliac*	0 (0%)	0 (0%)	1 (2.2%)	1 (0.4%)
* Urticarial Vasculitis*	0 (0%)	0 (0%)	1 (2.2%)	1 (0.4%)
* Myasthenia Gravis*	0 (0%)	1 (0.7%)	0 (0%)	1 (0.4%)
**Patients with >1 non-thyroid AI condition**	2 (2.8%)	5 (3.4%)	1 (2.2%)	8 (3.0%)
**Patients with one non-thyroid AI condition**	10 (13.6%)	12 (8.2%)	7 (15.2%)	29 (10.9%)
**Use of immunosuppressive therapy prior to TED symptom onset**	6 (8.1%)	7 (4.8%)	3 (6.5%)	16 (6.0%)

The presence of non-thyroid AI conditions was recorded if the condition had been diagnosed by a specialist following relevant examinations and investigations and reported in the patient’s medical records. Data presented as “count (percentage of cohort)”.

In total, 6.0% patients (16/267) underwent immunosuppressive therapy for their OAI conditions before TED symptom onset with no statistically significant differences between the ethnic groups. On average, immunomodulatory therapy for OAI conditions was commenced 60 (2–120) months prior to TED symptom onset; only 18.8% (3/16) of patients were still receiving immunosuppressive therapy at the time of TED diagnosis. Treatment of TED included steroids, monoclonal antibodies and disease modifying drugs. 31.3% (5/16) of patients used prednisolone prior to TED symptom onset, 31.3% (5/16) had taken monoclonal antibodies, namely natalizumab, alemtuzumab or tocilizumab, and 50.0% (8/16) had commenced disease modifying therapies, such as methotrexate, prior to TED symptom onset.

**Table 2** describes the characteristics of patients within three groups: those with TED only, those with TED + OAI conditions who had received immunosuppression for their AI conditions prior to TED symptom onset and those with OAI who had not been immunosuppressed. Significant differences were observed in the median age of presentation to MDT-TED clinic between the three groups. The median age of presentation to MDT-TED clinic of previously immunosuppressed patients was 52 (46.3–57.8) years, which was significantly higher than that of patients with TED only, 45 (33.8–54.0) years (*p =* 0.04). No differences were observed in the distribution of sex, ethnicity, or smoking status in TED-only patients compared to patients with OAI conditions, regardless of prior immunosuppression.

**Table 2:** Summary of patient demographics, thyroid dysfunction and thyroid eye disease (TED) data categorized by the presence of non-thyroid autoimmune conditions and whether or not patients with non-thyroid autoimmune conditions had commenced immunosuppressive therapy prior to TED symptom onset.

** **		TED only(n = 230)	TED + OAI conditions		p-value
			No Previous Immunosuppression(n = 21)	PreviousImmunosuppression(n = 16)	
***Patient Demographics***					
** Sex**	*Female*	182 (79.1%)	16 (76.2%)	14 (87.5%)	0.78
	*Male*	48 (20.7%)	5 (23.8%)	2 (12.5%)	
** Age at first MDT-TED clinic**		45 (34 - 54)	48 (43.5-61.5)	52 (44.8-57.8)	0.04*
** Smoking status**	*Current smoker*	54 (23.5%)	0 (0%)	2 (11.1%)	0.07
	*Previous smoker*	41 (17.8%)	3 (14.3%)	5 (27.8%)	
	*Vapes*	4 (1.7%)	1 (4.8%)	1 (5.5%)	
	*Never Smoked*	131 (57.0%)	17 (81.0%)	8 (50.0%)	
** Ethnicity**	*Caucasian*	62 (27.0%)	6 (28.6%)	6 (37.5%)	0.75
	*BAME*	127 (55.2%)	12 (57.1%)	9 (56.3%)	
	*Not Stated*	41 (17.8%)	3 (14.3%)	1 (6.2%)	
*** Thyroid Dysfunction***					
**Family history of thyroid dysfunction**		61 (26.5%)	3 (14.3%)	5 (27.8%)	–
** Diagnosed Thyroid Pathology**	*Graves*	200 (87.0%)	17 (80.1%)	15 (93.8%)	0.23
	*Hashimoto’s*	7 (3.0%)	2 (9.5%)	0 (0%)	
	*None*	4 (1.7%)	2 (9.5%)	1 (6.2%)	
	*Other*	2 (0.9%)	0 (0%)	0 (0%)	
** Thyroid status at TED onset**	*Hypothyroid*	18 (7.8%)	4 (19.0%)	0 (0%)	0.25
	*Euthyroid*	98 (42.6%)	8(38.1%)	9 (56.2%)	
	*Hyperthyroid*	114 (49.6%)	9 (42.9%)	7 (43.8%)	
** Previous Radioiodine Therapy**		32 (13.9%)	4 (19.0%)	1 (6.3%)	–
***Thyroid Eye Disease***					
** CAS at presentation**		1 (1-2)	2 (1-4)	1 (1-2)	0.04*
** Selenium recommended**		183 (79.6%)	20 (95.2%)	14 (87.5%)	–
** Proportion of patients requiring further therapy**	*IVMP only*	35 (15.2%)	2 (9.5%)	2 (12.5%)	–
	*IVMP + MMF*	25 (10.9%)	2 (9.5%)	0 (0%)	
	*IVMP + Radio*	18 (7.8%)	1 (4.8%)	1 (6.3%)	
	*IVMP + MMF + Radio*	9 (3.9%)	3 (14.3%)	0 (0%)	

Categoric data are presented as “count (percentage)”; all continuous variables are presented as “median (interquartile range).”

*represents statistical significance, where *p* < 0.05.

OAI, other autoimmune; TED, thyroid eye disease; BAME, black and minority ethnic; CAS, clinical activity score; IVMP, intravenous methylprednisolone; MMF, mycophenolate mofetil; Radio, orbital radiotherapy.

A similar proportion of TED-only patients (49.6%) and patients with OAI conditions (43.2%) presented to MDT-TED clinic with hyperthyroidism, regardless of prior use of immunosuppression. The proportion of patients that underwent radioiodine therapy for thyroid dysfunction was also comparable across the three groups of patients.

**Figure 1:**
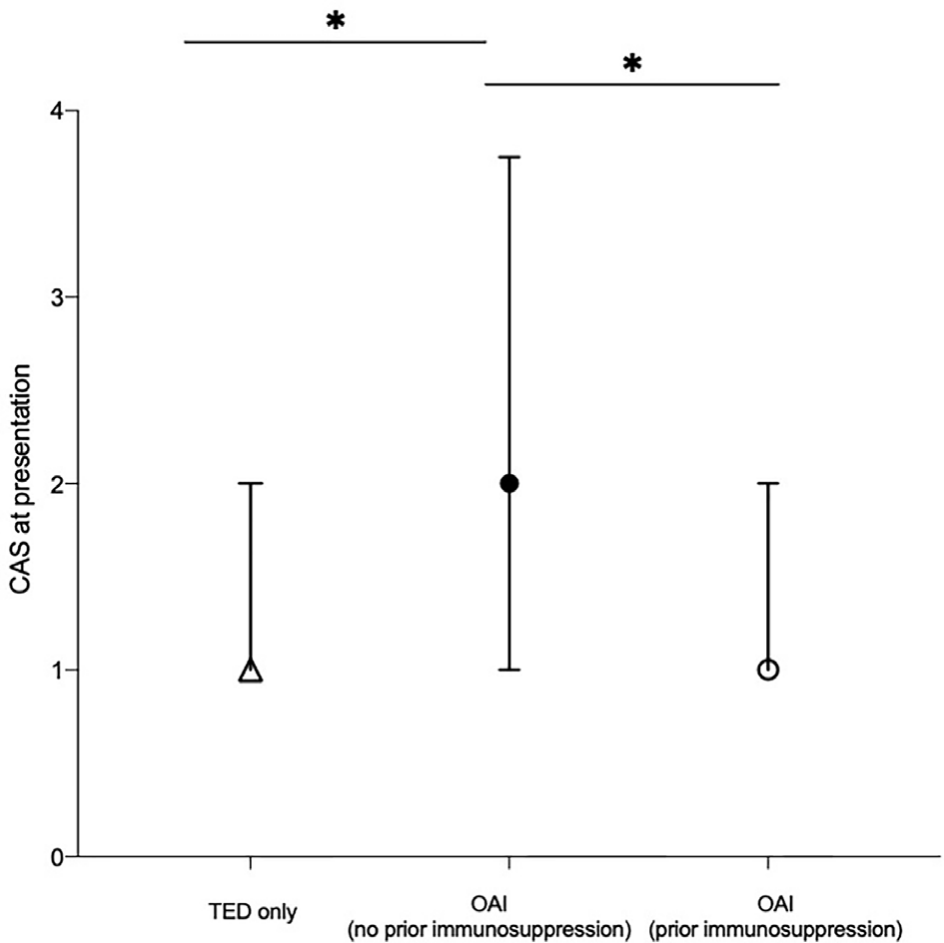
Median clinical activity score (CAS) at presentation of patients categorized by the presence of non-thyroid autoimmune conditions and whether or not patients with non-thyroid autoimmune conditions had commenced immunosuppressive therapy prior to TED symptom onset. Median value of each cohort plotted. Error bars represent the interquartile range. *represents statistical significance, where p < 0.0167 by Bonferroni’s correction. CAS, clinical activity score; OAI, other autoimmune; TED, thyroid eye disease.

Patients with OAI who had not received prior immunosuppressive therapy had the highest median CAS score at presentation (2, IQR: 1–4), compared to TED-only patients (*p =* 0.02; **Figure 1**) and previously immunosuppressed patients (*p* = 0.02; **Figure 1**).

Similar proportions of OAI (no prior immunosuppression) patients and OAI (prior immunosuppression) patients required first-line immunosuppressive therapy (IVMP) for TED (9.5% *vs.* 12.5%). However, only 6.3% of OAI (prior immunosuppression) patients required further (second-line) immunosuppressive TED therapy in the form of OR and/or MMF, while 28.6% of OAI (no prior immunosuppression) required first line (IVMP) and second line (OR and/or MMF) treatment.

### Thyroid Receptor Antibody (TRAb) Titer

**Figure 2:**
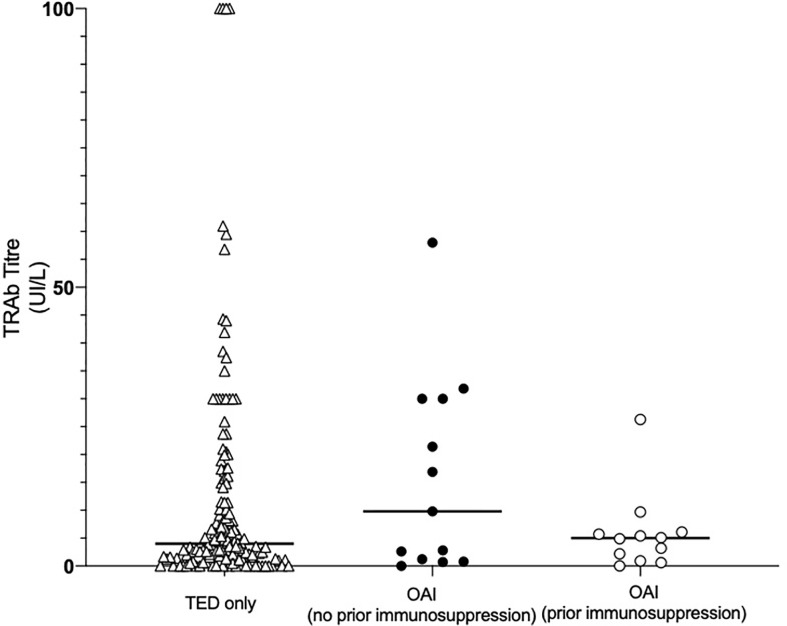
Initial thyroid receptor antibody (TRAb) titer of patients categorized by the presence of non-thyroid autoimmune conditions and whether or not patients with non-thyroid autoimmune conditions had commenced immunosuppressive therapy prior to TED symptom onset. Data were collected from clinic notes and patient hospital records. Points represent the initial TRAb titer of each patient in the cohort; solid lines represent the median TRAb titer of each cohort. No significant differences were found in the TRAb titers of the three groups. OAI, other autoimmune; TED, thyroid eye disease.

61.4% (164/267) of patients were tested for TRAb antibodies and 88.4% of these (145/164) were TRAb positive. There were no significant differences in the TRAb titers of patients with TED only compared to those with OAI, regardless of prior immunosuppression (*p* = 0.66; **Figure 2**). However, we note there was a tendency for OAI (no prior immunosuppression) patients conditions to have higher titers, 13.35 U/L (0.90–30.0), compared to OAI (prior immunosuppression) patients, 4.90 U/L (1.55–5.90), and those with TED only, 4.00 U/L (1.37–15.10; p>0.05).

Further sub-analysis found no correlation between TRAb titers and CAS of all included patients (*r* = 0.079, *p* = 0.32). There were also no correlations between TRAb and CAS within the three patient sub-groups.

We did not distinguish between stimulating and blocking TSH receptor antibodies as it was not possible to establish which assays had been used in the various laboratories across the three sites from 2011 to 2019.

## Discussion

This was the first study to evaluate the prevalence of non-thyroid OAI conditions in an ethnically diverse, metropolitan TED cohort and to assess potential associations between the presence of polyautoimmunity and TED severity or clinical activity.

The findings of this study, taken together with the results of the Cruz and Ferrari studies, suggest TED patients may have a higher prevalence of certain AI conditions compared to the general population. For example, RA and psoriasis was found to have a prevalence of 3.7% and 3% in our cohort, compared to a prevalence of 0.4%–1.2% and 1.3% in the general population (19). Our study also corroborates findings from the aforementioned studies that vitiligo and RA were among the most prevalent OAI conditions among TED patients. However, we also found a higher prevalence of psoriasis and MS in our cohort (16, 17).

A number of immune-related genes have been found in TED and OAI, presumably underpinning the inherited susceptibility to autoimmunity (20). There is evidence to suggest that mutations in the interleukin-23 receptor (IL23R) gene could confer increased susceptibility to AI conditions, such as RA, psoriasis and TED (21–23), which may explain the association of RA and psoriasis in our cohort. The link between IL23R gene mutations and TED pathophysiology is uncertain with only a few conflicting studies in the literature. For example, a study of Caucasian patients demonstrated the RA-associated single nucleotide polymorphisms of the IL23R gene to be associated with TED (24) while a similar study on Japanese patients did not identify such a trend (25). Further studies are needed to fully understand the role, if any, of the IL23R gene.

Vitiligo has previously been demonstrated to be associated with autoimmune thyroid diseases, such as Graves and Hashimoto’s thyroiditis (26–28). Similarly, autoimmune thyroid diseases have been demonstrated to be the most common co-existing AI conditions in MS and RA patients (29). It would be interesting to compare the prevalence of each AI conditions in patients with AITD and no orbitopathy to patients with TED to establish whether there is an association between TED and these AI conditions, or rather if the presence of AITD in the studied population was a confounder. Further studies are required on the potential value of screening with thyroid antibodies in patients with OAI conditions that appear to be most prevalent in those eventually presenting with TED.

We found 13.9% of patients to have at least one non-thyroid OAI condition, of whom 48.6% had prior immunosuppression before their TED diagnosis. Patients in the TED only and OAI (no prior immunosuppression) subgroups presented with more active and severe disease compare to the subgroup with OAI (prior immunosuppression) and required more immunosuppression for their TED. It is possible this finding is a chance association and further studies are required to confirm this.

This study found a higher proportion of euthyroid patients at presentation to TED-MDT compared to figures previously published in the literature (30). The nature of the referral pathway to these TED-MDTs is such that the majority of patients are referred from an endocrine clinic where therapy such as radioiodine, which is known to have an adverse effect on TED (30), may have been used to establish a euthyroid state.

A limitation of this study is that the term “immunosuppression” included patients who had taken monoclonal antibodies, disease modifying drugs or steroids prior to TED symptom onset. It is therefore unclear if the different mechanisms of action of various immunosuppressive agents could have different effects on TED severity or activity. Although only one patient had taken alemtuzumab, a recent study has demonstrated this drug to trigger the onset of TED in “at risk” patients (31). Whether other monoclonal therapies can have a similar effect is unknown. Beyond this, the effects of alemtuzumab on TED severity remain unclear. It would therefore be interesting to further study the effects of steroids, monoclonal antibodies and other disease modifying drugs on TED severity and activity. A further limitation of this study reflects this being a single-center, retrospective study. While our findings are comparable to the ethnic diversity of London, UK (BAME population of 40.2% as per the Office of National Statistics 2011 Census), the findings may not be representative of other parts in the UK (32). Furthermore, the study does not account for the effects of other medications, such as statins and metformin, among others, that could have an immunomodulatory properties (33).

Although the patients in this study were treated in the pre COVID-19 era, during the pandemic all first line intravenous steroid immunosuppression for non-sight threatening TED were stopped. We monitored and treated patients with severe relapse with oral steroids in line with best practice recommendations (34–36). All our second line immunosuppression patients continued treatment abiding with governmental shielding protocols. Careful consideration and constant evaluation has to be deployed regarding the choice and timing of immunosuppression in the post-COVID landscape.

In conclusion, the findings of this study were overall concordant with similar studies of monoethnic populations. Certain AI conditions, such as rheumatoid arthritis and vitiligo, appear to have an increased prevalence in TED patients. Whether or not this is due to a common pathway in their pathogeneses remains unclear. This study was also the first to demonstrate that patients with polyautoimmunity receiving immunosuppression prior to TED diagnosis may have less severe and less clinically active TED than those who have received no prior immunosuppressive therapy. Larger, prospective studies are warranted to evaluate how polyautoimmunity affects TED severity and activity and whether the presence of polyautoimmunity could be used as an early predictor of TED severity.

## Data Availability Statement

The raw data supporting the conclusions of this article will be made available by the authors, without undue reservation.

## Ethics Statement

The studies involving human participants were reviewed and approved by Imperial College Audit Office. Written informed consent for participation was not required for this study in accordance with the national legislation and the institutional requirements.

## Author Contributions

VL conceived and designed the study. MK acquired the data, analyzed, and interpreted the data. MK, PA, and VL drafted the manuscript. SF, RA, RJ, AA, CF, VB, and KM contributed to the manuscript. All authors contributed to the article and approved the submitted version. VL and MK are the guarantors of this work and, as such, had full access to all the data in the study and take responsibility for the integrity of the data and the accuracy of the data analysis.

## Conflict of Interest

The authors declare that the research was conducted in the absence of any commercial or financial relationships that could be construed as a potential conflict of interest.

## References

[1] Wiersinga WM Kahaly G. Graves" orbitopathy : a multidisciplinary approach. Karger (2007) p. 1–22. S. Karger (Firm). 10.1159/isbn.978-3-8055-8343-5

[2] Lazarus JH. Epidemiology of Graves’ orbitopathy (GO) and relationship with thyroid disease. Best Pract Res Clin Endocrinol Metab (2012) 26(3):273–9. 10.1016/j.beem.2011.10.005 22632364

[3] Vanderpump MPJ. The epidemiology of thyroid disease. Br Med Bull (2011) 99(1):39–51. 10.1093/bmb/ldr030 21893493

[4] Vangheluwe O Ducasse A Vaudrey C Maes B Delisle MJ. Prevalence de l’ophtalmopathie dans la maladie de basedow. Suivi des malades a un an apres la decouverte de leur hyperthyroidie. J Fr Ophtalmol (1994) 17(5):331–8. 8089419

[5] Bartley GB Fatourechi V Kadrmas EF Jacobsen SJ Ilstrup DM Garrity JA. Long-term follow-up of Graves ophthalmopathy in an incidence cohort. Ophthalmology (1996) 103(6):958–62. 10.1016/S0161-6420(96)30579-4 8643255

[6] Park JJ Sullivan TJ Mortimer RH Wagenaar M Perry-Keene DA. Assessing quality of life in Australian patients with Graves’ ophthalmopathy. Br J Ophthalmol (2004) 88(1):75–8. 10.1136/bjo.88.1.75 PMC1771927 14693779

[7] Ponto KA Merkesdal S Hommel G Pitz S Pfeiffer N Kahaly GJ. Public health relevance of graves’ orbitopathy. J Clin Endocrinol Metab (2013) 98(1):145–52. 10.1210/jc.2012-3119 23185037

[8] Mellington FE Dayan CM Dickinson AJ Hickey JL MacEwen CJ McLaren J. Management of thyroid eye disease in the United Kingdom: A multi-centre thyroid eye disease audit. Orbit (2017) 36(3):159–69. 10.1080/01676830.2017.1280057 28296512

[9] Bliddal S Nielsen CH Feldt-Rasmussen U. Recent advances in understanding autoimmune thyroid disease: The tallest tree in the forest of polyautoimmunity. F1000Research (2017) 6:1766. 10.12688/f1000research.11535.1 PMC5621109 29043075

[10] Agnoli A Ruggieri S Denaro A Bruno G. New Strategies in the Management of Parkinson’s Disease: A Biological Approach Using a Phospholipid Precursor (CDP-Choline). Neuropsychobiology (1982) 8(6):289–96. 10.1159/000117914 7162583

[11] Cellini M Santaguida MG Stramazzo I Capriello S Brusca N Antonelli A. Recurrent Pregnancy Loss in Women with Hashimoto’s Thyroiditis with Concurrent Non-Endocrine Autoimmune Disorders. Thyroid (2020) 30(3):457–62. 10.1089/thy.2019.0456 31910128

[12] Ramos-Casals M Nardi N Lagrutta M Brito-Zerón P Bové A Delgado G. Vasculitis in Systemic Lupus Erythematosus. Medicine (Baltimore) (2006) 85(2):95–104. 10.1097/01.md.0000216817.35937.70 16609348

[13] Christensen PB Jensen TS Tsiropoulos I SOrensen T Kjaer M Højer-Pedersen E. Associated autoimmune diseases in myasthenia gravis A population-based study. Acta Neurol Scand (2009) 91(3):192–5. 10.1111/j.1600-0404.1995.tb00432.x 7793234

[14] Marinó M Ricciardi R Pinchera A Barbesino G Manetti L Chiovato L. Mild Clinical Expression of Myasthenia Gravis Associated with Autoimmune Thyroid Diseases*. J Clin Endocrinol Metab (1997) 82(2):438–43. 10.1210/jcem.82.2.3749 9024233

[15] Avouac J Airò P Dieude P Caramaschi P Tiev K Diot E. Associated autoimmune diseases in systemic sclerosis define a subset of patients with milder disease: Results from 2 large cohorts of European Caucasian patients. J Rheumatol (2010) 37(3):608–14. 10.3899/jrheum.090815 20110522

[16] Cruz AAV Akaishi PMS Vargas MA de Paula SA. Association Between Thyroid Autoimmune Dysfunction and Non-Thyroid Autoimmune Diseases. Ophthalmic Plast Reconstr Surg (2007) 23(2):104–8. 10.1097/IOP.0b013e318030b06b 17413622

[17] Ferrari SM Fallahi P Ruffilli I Elia G Ragusa F Benvenga S. The association of other autoimmune diseases in patients with Graves’ disease (with or without ophthalmopathy): Review of the literature and report of a large series. Autoimmun Rev (2019) 18:287–92. 10.1016/j.autrev.2018.10.001 30639646

[18] Leo M Menconi F Rocchi R Latrofa F Sisti E Profilo MA. Role of the underlying thyroid disease on the phenotype of graves’ orbitopathy in a tertiary referral center. Thyroid (2015) 25(3):347–51. 10.1089/thy.2014.0475 25584927

[19] Symmons D Turner G Webb R Asten P Barrett E Lunt M. The prevalence of rheumatoid arthritis in the United Kingdom: new estimates for a new century. Rheumatology (2002) 41(7):793–800. 10.1093/rheumatology/41.7.793 12096230

[20] Franco J-S Amaya-Amaya J Anaya J-M. Thyroid disease and autoimmune diseases. (2013). https://www.ncbi.nlm.nih.gov/books/NBK459466/.

[21] Faragó B Magyari L Sáfrány E Csöngei V Járomi L Horvatovich K. Functional variants of interleukin-23 receptor gene confer risk for rheumatoid arthritis but not for systemic sclerosis. Ann Rheum Dis (2008) 67(2):248–50. 10.1136/ard.2007.072819 17606463

[22] Cargill M Schrodi SJ Chang M Garcia VE Brandon R Callis KP. A large-scale genetic association study confirms IL12B and leads to the identification of IL23R as psoriasis-risk genes. Am J Hum Genet (2007) 80(2):273–90. 10.1086/511051 PMC1785338 17236132

[23] Duerr RH Taylor KD Brant SR Rioux JD Silverberg MS Daly MJ. A genome-wide association study identifies IL23R as an inflammatory bowel disease gene. Science (80- ) (2006) 314(5804):1461–3. 10.1126/science.1135245 PMC4410764 17068223

[24] Huber AK Jacobson EM Jazdzewski K Concepcion ES Tomer Y. Interleukin (IL)-23 Receptor Is a Major Susceptibility Gene for Graves’ Ophthalmopathy: The IL-23/T-helper 17 Axis Extends to Thyroid Autoimmunity. J Clin Endocrinol Metab (2008) 93(3):1077–81. 10.1210/jc.2007-2190 PMC2266952 18073300

[25] Ban Y Tozaki T Taniyama M Nakano Y Yoneyama KI Ban Y. Association studies of the IL-23R gene in autoimmune thyroid disease in the Japanese population. Autoimmunity (2009) 42(2):126–30. 10.1080/08916930802422265 19021011

[26] Gey A Diallo A Seneschal J Léauté-Labrèze C Boralevi F Jouary T. Autoimmune thyroid disease in vitiligo: multivariate analysis indicates intricate pathomechanisms. Br J Dermatol (2013) 168(4):756–61. 10.1111/bjd.12166 23253044

[27] Gill L Zarbo A Isedeh P Jacobsen G Lim HW Hamzavi I. Comorbid autoimmune diseases in patients with vitiligo: A cross-sectional study. J Am Acad Dermatol (2016) 74(2):295–302. 10.1016/j.jaad.2015.08.063 26518171

[28] Sheth VM Guo Y Qureshi AA. Comorbidities associated with vitiligo: A ten-year retrospective study. Dermatology (2014) 4):311–5. 10.1159/000354607 24107643

[29] Rojas-Villarraga A Amaya-Amaya J Rodriguez-Rodriguez A Mantilla RD Anaya J-M García-Carrasco M. Introducing Polyautoimmunity: Secondary Autoimmune Diseases No Longer Exist. Autoimmune Dis (2012) 2012. 10.1155/2012/254319 PMC3290803 22454759

[30] McAlinden C. An overview of thyroid eye disease. Eye Vis (2014) 1(1):1–4. 10.1186/s40662-014-0009-8 PMC4655452 26605355

[31] Roos JCP Moran C Chatterjee VK Jones J Coles A Murthy R. Immune reconstitution after alemtuzumab therapy for multiple sclerosis triggering Graves’ orbitopathy: a case series. Eye (2019) 33(2):223–9. 10.1038/s41433-018-0282-1 PMC6367353 30498266

[32] Regional ethnic diversity. GOV.UK Ethnicity facts and figures. https://www.ethnicity-facts-figures.service.gov.uk/uk-population-by-ethnicity/national-and-regional-populations/regional-ethnic-diversity/latest.

[33] Koch CA Krabbe S Hehmke B. Statins, metformin, proprotein-convertase-subtilisin-kexin type-9 (PCSK9) inhibitors and sex hormones: Immunomodulatory properties? Rev Endocr Metab Disord (2018) 19:363–95. 10.1007/s11154-018-9478-8 30673921

[34] Bartalena L Chiovato L Marcocci C Vitti P Piantanida E Tanda ML. Management of Graves’ hyperthyroidism and orbitopathy in time of COVID-19 pandemic. J Endocrinol Invest (2020) 43(8):1149–51. 10.1007/s40618-020-01293-7 PMC7241069 32441005

[35] Schön MP Berking C Biedermann T Buhl T Erpenbeck L Eyerich K. COVID-19 and immunological regulations – from basic and translational aspects to clinical implications. JDDG J der Dtsch Dermatologischen Gesellschaft (2020) 18: (8):795–807. 10.1111/ddg.14169 PMC7436872 32761894

[36] TED Immunosuppression advice during Covid-19 – BOPSS. Available at: https://www.bopss.co.uk/covid/ted-immunosuppression-advice-during-covid-19.

